# Tobacco

**Published:** 1880-08

**Authors:** 


### TOBACCO.

The following from one of our daily papers, gives an inkling as to how the tobacco habit appears to some people.

Complaint has been made by women that as they go along the sidewalk they are defiled by spittle and spent quids of tobacco from the windows above. They who have observed the habits of tobacco chewers have noticed that they spit and throw quids from windows without any thought of the passers. This is strictly logical. He who makes his mouth nasty can not have any sensation at nastiness anywhere. Logically, a person can not have any higher regard for cleanliness in outward objects, whether person or things, than he has for his own mouth. He that makes himself nasty is by the force of logic led to defile all others. The tobacco chewer is inevitably demoralized in all his manners.

